# Clot formation, structure, and fibrinolysis of pancreatic cancer patients

**DOI:** 10.21203/rs.3.rs-5868575/v1

**Published:** 2025-02-11

**Authors:** Rebecca A Risman, Noam Milman, Hajer Sinan, Valerie Tutwiler

**Affiliations:** Rutgers, The State University of New Jersey

**Keywords:** fibrin, fibrinogen, fibrinolysis, pancreatic cancer, plasminogen activator inhibitor 1, thrombosis

## Abstract

**Background:**

Pancreatic cancer (PC) has the highest risk of venous thromboembolisms amongst all cancer types. If not degraded through a process known as fibrinolysis, thrombi will continue to restrict blood flow and the transport of nutrients to downstream organs, which can lead to heart attack or stroke. While PC patients are known to be hypercoagulable and thus have an elevated thrombosis risk, the mechanism behind this behavior is not fully understood.

**Aims:**

We aimed to characterize alterations in clotting and fibrinolytic profiles in PC patients compared to healthy controls.

**Methods:**

Human blood plasma was collected from PC patients and healthy donor controls following institutional review board approval. We used kinetic turbidity to define the rates/timing of blood clot formation/degradation. Confocal and scanning electron microscopy were used to probe the effect PC has on fibrin network structure. Concentrations of proteins for clotting/fibrinolytic pathways were measured using ELISAs.

**Results:**

PC patients were hypercoagulable compared to healthy donors with heightened fibrinogen concentration. A subset of patients were hypofibrinolytic, while most had similar fibrinolytic profiles to healthy. A comprehensive analysis revealed that delayed lysis in this subset was only present in patients with diabetes and/or COVID-19 due delayed clotting and, notably, elevated plasminogen activator inhibitor (PAI-1). In the general PC population, an extended PTT correlated with thicker fiber diameters while faster clotting resulted in smaller network pore size but was not correlated with lysis rate. Healthy, pooled plasma spiked with relevant concentrations of PAI-1 showed no difference in clot structure and comparable delays in lysis to patients.

**Conclusion:**

PAI-1, rather than network structure or other clotting/fibrinolytic factors, played a more significant role in hypo fibrinolysis. PAI-1 inhibitors could be a prospective target for development of improved therapeutics to prevent restricted fibrinolysis.

## INTRODUCTION

Pancreatic cancer (PC) has the highest risk of venous thromboembolisms (VTE) of all cancer types with 20% of PC patients experiencing a VTE, resulting in elevated risk of heart attack or stroke [1–8]. PC is a deadly cancer with a 5-year survivor rate of 10%. The formation of a VTE reduces the quality of life, increases the cost of health care needs, and increases the risk of mortality [6]. The pancreatic tumor microenvironment is a complex system that affects all aspects of the body, including hemostasis [9]. Moreover, standard chemotherapies, such as cisplatin, exacerbate the patient’s hypercoagulability, making the patient more susceptible to blood clot formation [10, 11]. Prophylactically, it is recommended to prescribe anticoagulants to PC patients to reduce the risk of VTE [12]. However, this proactive treatment plan can lead to a risk of bleeding during tumor resection surgery [13, 14]. Therefore, it is essential that a more complete coagulation profile of the PC patient population is defined to accurately predict risk of and therapeutic needs for thrombotic complications.

Thrombosis occurs when a blood clot forms within the vessel and prevents the flow of nutrients that can result in organ damage, heart attack, or stroke. The clotting cascade is initiated when platelets become activated and tissue factor (TF) is released from the damaged endothelial cells at the blood vessel injury site. This ultimately leads to the conversion of prothrombin into thrombin. Thrombin cleaves sites on fibrinogen, a blood plasma protein, which polymerizes into a fibrin network [15]. Fibrin provides the structural and mechanical stability to blood clots [16, 17]. To restore blood flow, the clot must degrade through a process known as fibrinolysis. Fibrinolysis occurs when circulating tissue plasminogen activator (tPA) converts plasminogen to plasmin which interacts with the fibrin networking, breaking it down into fibrin degradation products [18]. Hemostasis is the careful balance between clotting activators (i.e., thrombin or TF), clotting inhibitors (i.e., anticoagulants), fibrinolytic agents (i.e., tPA), and fibrinolytic inhibitors. Some key fibrinolysis inhibitors include plasminogen activator inhibitor (PAI-1), which irreversibly binds and inactivates tPA; thrombin activatable fibrinolytic inhibitor (TAFI) is activated via thrombin and cleaves the plasmin binding site on a fiber; tissue factor pathway inhibitor (TFPI) inhibits thrombin generation. All inhibitors have been studied in diseased conditions, including COVID-19 and polycystic ovary syndrome [19–22]. Notably, levels of PAI-1 in PC *in vitro* and *in vivo* models was correlated to the risk of VTE [23–25]. However, patient studies measuring PAI-1 have contradicting results in the inhibitor’s role in VTE. An older study from 1992 found elevated PAI-1 in PC patients with deep vein thrombosis [23]. A more recent study in 2018 did not find a correlation between PAI-1 and VTE occurrence [26]. Understanding the role of PAI-1 in PC and risk of VTE can aid in the management of thrombosis risk. Studying the changes that occur in PC is especially important due to the elevated risk of thrombosis and can be used to inform cancer associated thrombosis more broadly.

It is well known in the field of fibrin(ogen) and clotting research that fibrin network structure affects susceptibility to lysis; notably, the network density and fiber diameter play a significant role [27–29]. For example, COVID-19 patients had higher fibrin network densities compared to healthy donors and restricted lysis *in vitro,* but this did not correlate to risk of thrombosis [30]. However, there has been limited intersectionality that explored the fibrin network structure in cancer-associated thrombosis. Some work has identified that chemotherapy treatment in lung cancer does not affect network structure; however, they did find a greater permeability in patients who did not smoke [31]. The fibrin network profile for diseases such as PC have yet to be defined [32].

While the hypercoagulability of PC patients has long been established both clinically and in basic science, the fibrinolytic profile has yet to be understood [23, 33]. Recently, a study characterized the clotting properties of PC patients and observed in their optimized *in vitro* assay that PC patients have increased rate of clot formation and higher absorbance. They speculated, but did not characterize, the role these results played in fibrin network structure nor how it would affect susceptibility to lysis [34]. The clots that form (i.e., the structure) under hypercoagulable conditions with PC patients have not been characterized as well as how they affect the ability or inability for a clot to degrade [35]. Understanding the interplay between altered expression levels of coagulation factors and pro-/anti-fibrinolytic factors can lead to greater insight into pro-coagulation and hypo fibrinolysis in cancer patients. This research could reveal the mechanisms exhibited by PC plasma that heightens thrombosis risk and complicates combination treatment with chemotherapy and anticoagulants. Ultimately, this could help develop better diagnostics to predict or therapeutics needed to target these pathways. In the present pilot study, we explore the coagulation profile of PC patients through the employment of kinetic assays, structural techniques, and protein composition in the plasma.

## METHODS

### Patient sample collection

Blood samples were collected from PC patients (n=17) at the Cancer Institute of New Jersey following informed consent under approval by the Rutgers University Institutional Review Board (Pro2022001970). Deidentified patient data was retrieved, including comorbidities, chemotherapy, and anticoagulants at the time of blood draw, as well blood clotting events within 12 months before the blood draw (earliest blood draw was 2022) until December 2024 (Table 1). Labs, such as complete blood count (CBC) was analyzed, which included prothrombin time (PT) and partial thromboplastin time (PTT). Blood was drawn into sodium citrate tubes. Whole blood was centrifuged at 2500 RPM to isolate plasma. Extracted plasma was stored at −80°C.

### Healthy donor sample collection

Blood samples were collected from healthy donors (n=7) following informed consent under approval by the Rutgers University Institutional Review Board (Pro2020001694). Blood was collected into sodium citrate tubes. Whole blood was centrifuged at 2400 RPM to isolate plasma. Extracted plasma was stored at −80°C.

### Kinetic tracking of clot formation and fibrinolysis

Plasma samples were warmed at 37°C until thawed for at least 20 minutes, and diluted with buffer (1x HEPES Buffered Saline, Sigma cat# 51558) to form a 30% clot by volume [36]. Samples were activated with calcium chloride (final concentration of 25 mM, Sigma 223506), and phospholipids (final concentration 4 μM, Rossix, Cat no: PL604) to replicate what was previously done in similar studies [34]. Supplemental results show activation of clotting with 1pM tissue factor (TF) instead of phospholipids (Supplemental Figure 1). Tissue plasminogen activator (tPA) (Sigma 612200, final concentration 40 ng/mL) was added to the plasma before clot formation to initiate lysis. The mixture of diluted plasma, tPA, CaCl_2_, and phospholipids were added to each well of a 96-well plate in technical replicates as previously described [36]. The samples were surrounded by water wells to create a humid chamber to prevent plasma clots from drying. The well plate was then inserted into a Molecular Devices SpectraMax iD3 and the optical density (OD) was measured at 405 nm in intervals of 15 seconds for a duration of six hours. Experiments were performed in triplicate.

Turbidity data was analyzed for five parameters: 1) the time it took to initiate clotting (clotting lag time), 2) maximum optical density (max OD), 3) rate of formation, 4) time to 50% lysis, and 5) rate of lysis. The raw data, normalized to 0, gave us when each curve reached its max OD. To effectively analyze rate of formation and lysis, the clot lysis curves were normalized to its maximum point, showing the fraction of clot when fully formed as 1 and fully lysed clots as 0. The lag time was the point at which 5% of the clot had formed (0.05 fraction of clot) while the time to 50% lysis was the time at which the clot decreased to 0.5 fraction of clot. The rate of formation was characterized as the slope of the linear region of the curves from 20% clot to 80% clot while the rate of degradation was characterized as the slope after the maximum from 80% clot to 20% clot [37].

### Confocal microscopy

Samples were made in the same manner as for turbidity experiments with the exclusion of tPA. Samples were labeled with 1% (by volume of plasma volume) of a fluorescent conjugate for fibrinogen (Alexa Fluor 594, Invitrogen F13191). Images were captured using a Zeiss 780 confocal microscope with 40x magnification with a water objective and numerical aperture of 1.20 (1024×1024 pixels). Maximum intensity projections (MIPs) of representative z-stacks (11 slices – range of 10 μm, 1 μm interval) were processed for three locations of each sample; two samples were imaged for each day, three images per sample. Images were analyzed on FIJI ImageJ for pore size and percent area of fibers. Pore sizes were manually measured by overlaying a grid and measuring the distance between fibers at each intersection of the grid, resulting in 64 measurements of each image, as previously described [38].

### Scanning electron microscopy

For scanning electron microscopy (SEM), samples were formed with the same conditions outlined for turbidity experiments, with the exclusion of tPA, and prepared as previously reported [38]. Briefly, the samples were washed in sodium cacodylate buffer (Electron Microscopy Sciences 11652), fixed in 2% glutaraldehyde in sodium cacodylate buffer overnight, and washed again in sodium cacodylate buffer. The samples were then dehydrated with diluted 200 proof EtOH of increasing concentrations from 30% to 100% and finally chemically dried with hexamethyldisilazane (HMDS, Electron Microscopy Sciences 16700) until evaporation. After this dehydration process, the samples were mounted on carbon tape on stub holders, sputter coated with 10 nm of gold/palladium (80/20) and imaged using a Phenom Desktop SEM at 30k magnification. A grid was overlaid to measure the diameter using FIJI. At least 50 fibers were measured for six images per patient replicates [38].

### Measurements of clotting/fibrinolytic activators and inhibitors

#### Fibrinogen:

The fibrinogen concentration was determined using a Stago STart 4 in which diluted plasma was activated with Dade Thrombin Reagent (Siemens Healthineers, Cat #10445720). The movement of a metal ball was tracked until it stopped moving, in which this was recorded as the prothrombin time. A log-log graph was plotted with a standard curve of plasma with a known concentration (Standard Human Plasma, Siemens Healthineers, Cat #23-044-735) to calculate the fibrinogen concentration in the patients and healthy donors. Samples were performed in duplicate.

#### Thrombin:

Thrombin generation was measured using a commercially available thrombin generation assay (DiaPharma, Catalog #5006010). Fluorescence (RFU) was measured using a plate reader to quantify generation. Thrombin generation was calculated using the provided equation sheet and a standard curve with known concentrations. Samples were performed in duplicate.

#### PAI-1 and TAFI:

Commercially available enzyme linked immunosorbent assays (ELISAs) were performed to measure the concentrations of PAI-1 (Abcam Cat#ab157528) and TAFI (Abcam Cat#ab272774). Plasma samples were warmed to 37°C until thawed. Manufacturer’s procedures were followed for preparation. Concentrations in patient plasma were calculated using a standard curve with known concentrations. Samples were performed in duplicate.

### Commercial plasma spiked with PAI-1

#### Sample preparation and turbidity

Commercially available platelet-poor, human pooled plasma with fibrinogen concentration of 2.9 mg/mL was used to create plasma clots for turbidity experiments (Cone Bioproducts # 5781, pooled source human plasma). Plasma samples were warmed at 37°C and diluted with buffer for a 30% clot by volume, as done with the PC patients above (50 mM Tris (Sigma 648315), 140 mM NaCl, 1 mg/mL BSA) (final fibrinogen concentration of 0.70 mg/mL), tPA (Sigma 612200, final concentration 40 ng/mL) and varying concentrations of exogenous PAI-1 (Sigma A8111, final concentrations of 0, 10, 50, 100, and 300 ng/ml). Control samples were formed the same way but without tPA. Samples were activated with calcium chloride (final concentration of 25 mM, Sigma 223506), and thrombin (Sigma T1063, final concentration of 0.1 U/mL). Thrombin was used to mimic the hypercoagulable environment of PC patients. The plasma solution and activation mix were added to each well of a 96-well plate in technical replicates. The samples were surrounded by water wells to create a humid chamber to prevent plasma clots from drying. The well plate was then inserted into a SpectraMax Microplate Reader and the optical density (OD) was measured at 405 nm in intervals of 15 seconds for a duration of 8 hours. Experiments were performed on three different days (N=3) in triplicate (n=9). The time to 50% lysis was recorded as defined previously.

#### Confocal microscopy

Samples were made in the same manner as for turbidity experiments with the exclusion of tPA with a fluorescent conjugate for fibrinogen (Alexa Fluor 488, Invitrogen F13191). Images were captured using a Zeiss 780 confocal microscope with 40x magnification with a water objective and numerical aperture of 1.20 (1024×1024 pixels, resolution of 203 nm). Images were taken on three different days (N = 3) with two samples per day and three images per sample (n = 18). Maximum intensity projections (MIPs) of representative z-stacks (31 slices – range of 30 μm, 1 μm interval) were processed for three locations of each sample. Images were analyzed on FIJI ImageJ for branching and fibrin density. Fibrin density was measured as described above.

### Statistical analysis

Statistical analysis was completed using GraphPad Prism 10.0. All data is represented as mean ± SEM (standard error of the mean) unless otherwise noted. Statistical outliers were removed with Grubs’ test with α=0.05. Normality was checked using the D’Agostino and Pearson test with a significance level of p<0.05. Unpaired t-tests were performed to compare PC vs healthy. Multiple comparisons tests were analyzed using adjusted one-way ANOVA tests to account for significantly different variances (Brown-Forsythe ANOVA test) or non-normal distributions (Kruskal Wallis). Pearson correlation tests were performed to compare parameters.

## RESULTS

### I. Pancreatic cancer patients have altered clotting and lysis profiles.

Clotting and fibrinolysis was measured from PC patients and healthy donors (Figure 2a). Plasma from PC patients clots earlier and faster as seen by shorter lag times (Figure 2b, p<0.01) and faster rate of formation (Figure 2c, p<0.01) when compared to healthy controls. This confirmed previous studies [34]. PC patients had a slightly higher maximum optical density, but insignificantly different compared to healthy controls (Figure 2d, ns). While there was no difference in time to 50% lysis (Figure 2e, ns) and degradation rate (Figure 2f, ns), there was a subset of patients that took longer to lyse or did not lyse within the experimental period. Slower rate of degradation was correlated to longer time to 50% lysis (Supplemental Figure 2a). There was a correlation between clotting lag time and fibrinolytic profile — the clots that were resistant to lysis had delayed clot initiation (Supplemental Figure 2b, c). Similarly, a prolonged partial thrombin time (PTT) in a subset of patients in which this parameter was measured, the longer it took for the *in* vitro clot to degrade (Supplemental Figure 2d).

While there was not a direct relationship between PC and time to 50% lysis, identifying comorbidities revealed that some patients are more susceptible to fibrinolytic resistance. In particular, PC patients who also were diagnosed with diabetes (n=7), COVID (n=2), or hypertension (n=10) at the time of the blood draw, made up the population of fibrinolytic resistance in our *in vitro* assay (Figure 2g, h).

### II. Pancreatic cancer minimally affects clot structure.

We performed microscopy experiments to explore the role of PC on fibrin network structure (Figure 2a, b). Firstly, PC patients had a slightly, yet statistically significantly, smaller fibrin network pore sizes compared to the healthy controls (Figure 4c, p < 0.0001), indicating a denser network. However, there was no significant difference in the percent area of fibers per region of the clot between PC patients and healthy controls (Figure 4d, p > 0.05, ns). Although the difference in OD would suggest a change in fibrin network structure, pore size did not correlate to rate of formation, time to 50% lysis, or max OD (Supplemental Figure 3a-c). Different anticoagulants did not alter pore size (Supplementary Figure 3d).

Scanning electron microscopy images were used to analyze the diameters of the fibrin fibers in PC patients as compared to the healthy patients (Figure 3f, G). It was found that there is no difference in the fiber diameter (Figure 3h p > 0.05, ns). A delay in clotting did not correlate to thicker diameters (Supplemental Figure 3e). Fiber diameter was then compared to the maximum optical density, and it was shown that higher fiber diameter correlated with a larger maximum optical density (Supplemental Figure 3f). Fiber diameter had no effect on time to 50% lysis (Supplement Figure 3g). In a subset of patients, in which partial thrombin time (PTT) was measured, PTT corresponded to a thicker diameter (Supplemental Figure 3h).

### III. Modulation of clotting and fibrinolytic factors in pancreatic cancer patients.

To understand the driving force behind hypercoagulability in PC patients, we assessed coagulation factor concentrations. Thrombin generation was lower for PC patients than in healthy donors (Figure 3a), which had been seen previously in plasma [39]. Patients who also had COPD (n=3) had more thrombin generation than patients without and slightly more than healthy donors (Figure 3b). PC patients had higher concentrations of fibrinogen relative to healthy controls (Figure 3c). Neither thrombin generation nor fibrinogen correlated to initiation or rate of clotting (Supplemental Figure 4a, B). An increase in fibrinogen concentration correlated to a smaller hematocrit (Supplemental Figure 4c), higher max OD (Supplemental Figure 4d), and thicker fiber diameter (Supplemental Figure 4d). Fibrinogen concentration and thrombin generation did not affect fibrinolysis (Supplemental Figure 4f, g).

**Figure 1:**
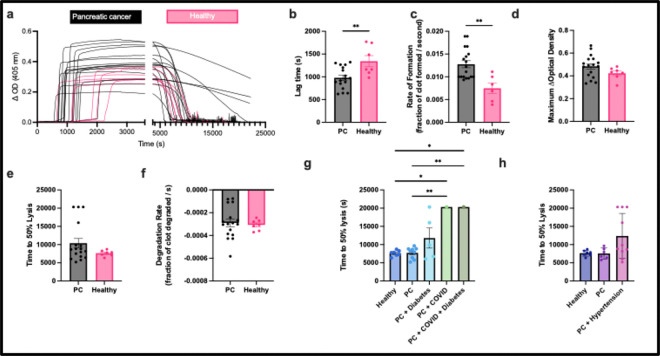
Clot formation and lysis of PC patients compared to healthy patients. a) Clotting and fibrinolysis kinetics of individual patients. One replicate (out of three) is shown due to inner variability. b) Lag time, c) maximum change in optical density, d) rate of formation, e) time to 50% lysis, and f) degradation rate was measured from turbidity. Role of g) diabetes, COVID, and h) hypertension on time to 50% lysis. * p<0.05, ** p<0.01.

To probe the mechanism for why the subset of patients were more resistant to lysis since it was not due to fibrin network structure or clotting factors, we assessed fibrinolytic inhibitor concentrations. Most patients had comparable concentrations of PAI-1 to healthy donors (1–20 ng/mL, Figure 4d). Of note, most hypofibrinolytic patients had diabetes or COVID-19 (Figure 4e) similar to as seen in Figure 1g. The subset of patients who were hypofibrinolytic were resistant to lysis as seen with turbidity (Figure 1) had a higher concentration of PAI-1 which was confirmed with a correlation test between PAI-1 and time to 50% lysis (Supplemental Figure 4h). Higher concentrations of PAI-1 correlated to elongated PTT (Supplemental Figure 4i). Although there were slightly lower levels of TAFI in PC patients compared to healthy controls, it was not significantly different nor was there a correlation between TAFI levels and time to 50% lysis (Figure 4d, Supplemental Figure 4j).

### IV. Plasma spiked with PAI-1 delayed fibrinolysis without altering clot structure.

Upon discovery that PAI-1 was the driving force behind restriction of lysis in a subset of PC patients, we sought to isolate the role of PAI-1. First, we identified that PAI-1 does not affect clot structure (Figure 6a-c) as seen with the percent area of fibers (Figure 6d, ns), as expected due to its exclusive role on the fibrinolysis side of the coagulation cascade. However, when we varied the PAI-1 concentration with exogenous PAI-1 (Figure 6e), we saw the expected delay in time to 50% lysis (Figure 4f, p<0.0001). These conditions had similar timing for low (50 ng/mL, “regular” PC patient) and high (300 ng/mL, “hypofibrinolytic” patient) PAI-1 concentrations (Figure 6g).

## DISCUSSION

Diseases such as cancer, COVID-19, and diabetes have an elevated risk of thrombosis. In particular, PC has the highest risk of VTE amongst all cancer types. While cancer-associated thrombosis has been long acknowledged, the mechanisms underlying this behavior as well as ideal prophylactic treatments and diagnostics have yet to be fully understood [33]. A more complete understanding of the individual contributions of factors that could affect a PC patients’ risk of thrombosis could improve quality of life and patient outcome. Many PC patients experience comorbidities that can compound the thrombotic effects of PC by itself. Similarly, people diagnosed with diabetes or prediabetes are more likely to develop PC [40]. Thus, it is unsurprising that PC patients in our study who also had prediabetes, diabetes, and/or COVID were more susceptible to restricted fibrinolysis in our *in vitro* assay (Figure 1G). The present study sought to identify the dominating feature that led to the hypercoagulability and hypo fibrinolysis of these PC patients. This could reveal a potential for a new, targeted treatment.

In the present study, we utilized a combination of experiments in which we activated the coagulation cascade using phospholipids and CaCl_2_ to mimic the conditions that Thaler et al established to show hypercoagulability of PC patients [34]. In the supplement, we show that TF activation results in a similar clotting profile but different fibrinolytic potential (Supplemental Figure 1). Turbidity is an easy and high throughput technique to quantify hypercoagulability of patients in which calculated parameters from turbidimetric curves have a correlation to PTT, fibrinogen concentration, and PAI-1 concentration (Supplemental Figures 2–4). Moreover, this experimental design only requires less than 300 μL of fresh or frozen patient plasma with and without tPA each with three replicates. Previously established protocols motivated by the International Society of Thrombosis and Haemostasis (ISTH) subcommittee initiated this conversation [36, 41], yet thrombin and TF can mask the affects in patient samples making it difficult to make predictions (Supplemental Figure 1) [34]. This highlights the need to establish universal techniques to be able to study patient samples and compare across laboratories [42]. This is particularly important for patients with PC, as Thaler et al also identified, to understand the mechanisms of hypercoagulability and hypo fibrinolysis. Moreover, experimental conditions, such as the addition of TF, confounded results and can limit conclusions (Supplemental Figure 1). In order to perform *in vitro* testing on potential therapeutics for PC patients, there is a need to develop a method that allows researchers to study coincubation of patient plasma with anticoagulants, chemotherapy, fibrinolytic inhibitors, and other factors relevant to PC. Due to the high throughput nature of this technique, the individual contributions of these treatments could be broadly characterized. Optimized and standardized techniques would make these simple protocols more acceptable and widely used for diagnostics and therapeutic management of cancer-associated thrombosis.

**Figure 2:**
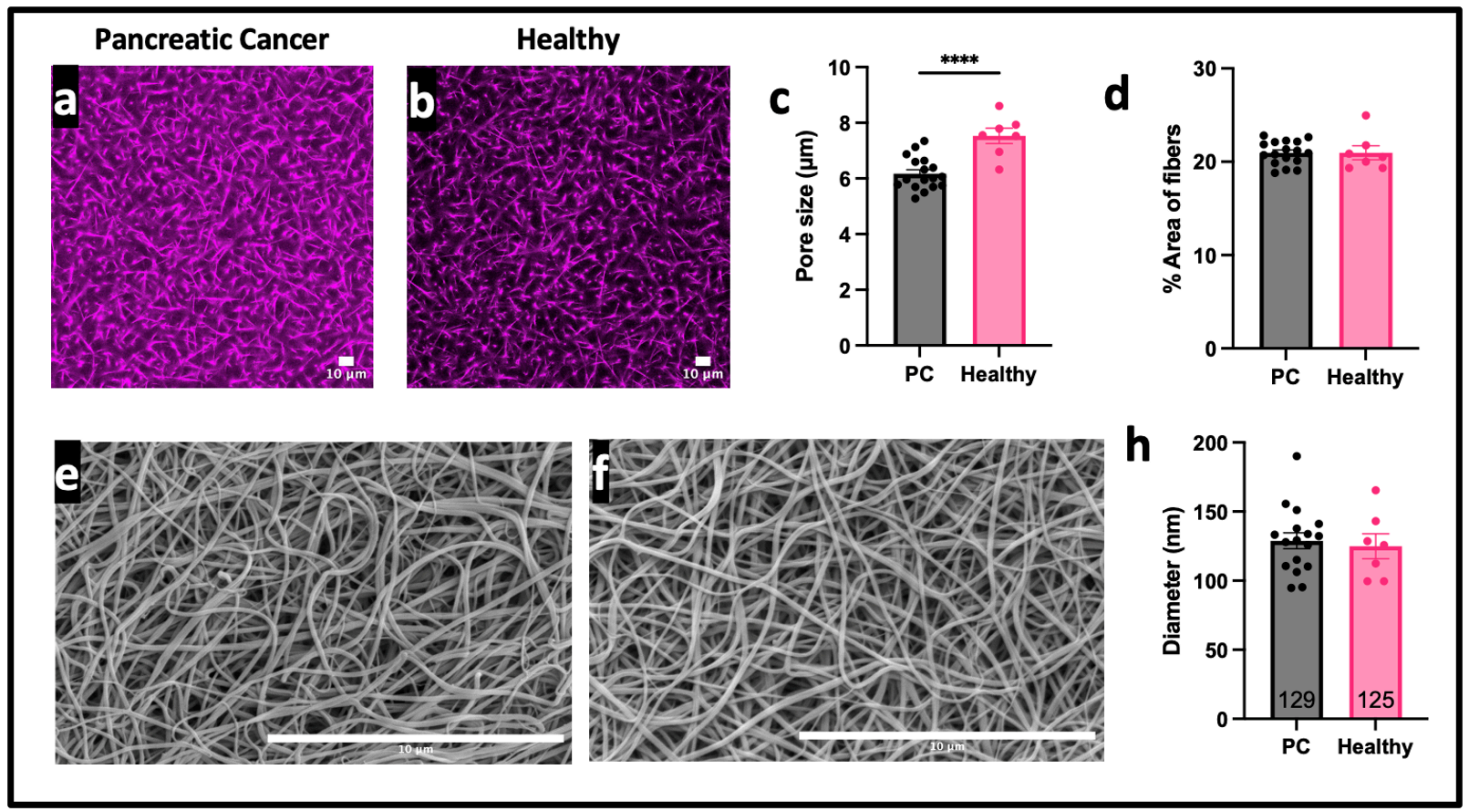
Clot structure of PC patients compared to healthy patients as seen with microscopy. Confocal microscopy images of fibrin clots made with PC patient (a) and healthy donor (b) plasma. Pore size (c) and percent area of fibers (d) were measured from the confocal images. Scanning electron microscopy images of fibrin clots made with PC patient (e) and healthy donor (f) plasma. Diameter (h) was measured from scanning electron microscopy images. Scale bar is 10 microns. **** p<0.001.

Due to an established notion that fibrin structure affects risk of VTE and resistance to fibrinolysis [18, 28, 29, 37, 43–45], we sought to characterize the fibrin clot structural profile of PC patient plasma samples compared to healthy donor controls. More specifically, elevated fibrinogen concentrations, as seen with hypercoagulable conditions, is known to produce thicker fibers and denser fibrin networks, which are associated with longer degradation times [37, 42]. Our results showed that PC patients have a denser network but no difference in fiber diameter (Figure 2). However, the pore size difference seen in the current study (~1.5 microns) was ~5x smaller than what we saw in our previous studies when we suggested an altered tPA affinity (~8 microns) [37]. It has previously been identified that diabetes and COVID-19 affect fibrin structure [30, 46], suggesting that PC may also have altered networks. More specifically, it has been shown that dense fibrin networks, with small pore sizes, limit lysis through restrictive permeability of tPA [44]. This could be due to less space for tPA to diffuse or perfuse through the fibrin network [37]. Alternatively, this could be due to tPA having too high of an affinity for fibrin, causing it to stay bound to a fiber for too long and unable to bind to and degrade new fibers [44]. Our previous work, along with others, suggested that a more effective tPA variant would have a lower affinity for fibrin, so it does not stick to fibers in a dense network [44, 47]. However, because the current study suggests that the fibrin network structure in PC does not affect fibrinolysis, further research to treat blood clots for PC patients does not have to focus on the tPA: fibrin ratio, tPA’s affinity for fibrin, or the ability of tPA to diffuse/perfuse.

Furthermore, it was previously shown that while apixaban and enoxaparin affect fibrin network structure, heparin minimally affects fiber diameter and permeability [38, 48, 49]. Since all the patients were on heparin, and only some were taking apixaban or enoxaparin at the time of blood draw, we can suspect that the anticoagulant was not affecting network structure. Moreover, the different anticoagulant treatments did not affect the pore size (Supplemental Figure 3d). Despite the slight difference in pore size (Figure 2c), neither pore size nor diameter correlated to restricted fibrinolysis (Supplemental Figure 3b, e). While the change in pore size seen in the present study was statistically significantly different between PC patients and healthy donors, it was not enough to affect lysis and should not be considered when developing improved therapeutics. Therefore, the remainder of the study sought to identify alternate factors that contributed to hypo fibrinolysis to aid in the design of more efficient anticoagulants or fibrinolytic agents.

**Figure 3:**
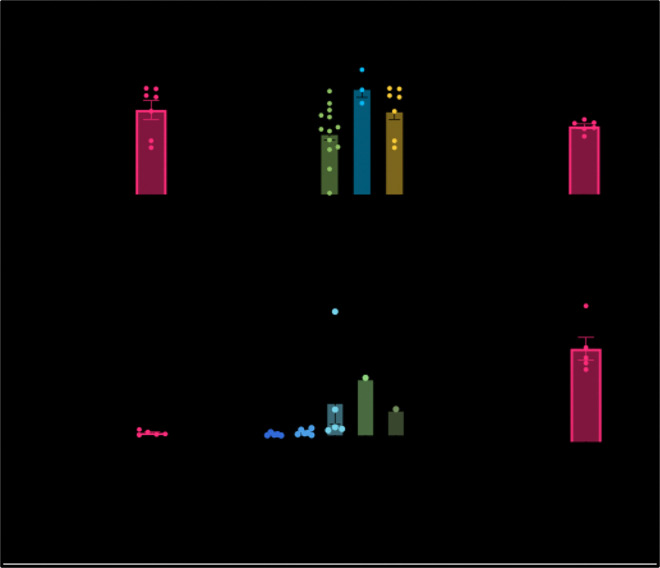
*Concentrations of clotting activators and inhibitors of PC patients compared to healthy patients. a) Thrombin generation of PC vs healthy. b) Thrombin generation of PC vs PC+* Chronic obstructive pulmonary disease (COPD) vs healthy. c) Fibrinogen concentration of PC vs healthy. d) PAI-1 concentration for PC vs healthy. e) PAI-1 concentration of PC vs with diabetes and/or COVID vs healthy. f) TAFI concentration of PC vs healthy. * p<0.05.

**Figure 4:**
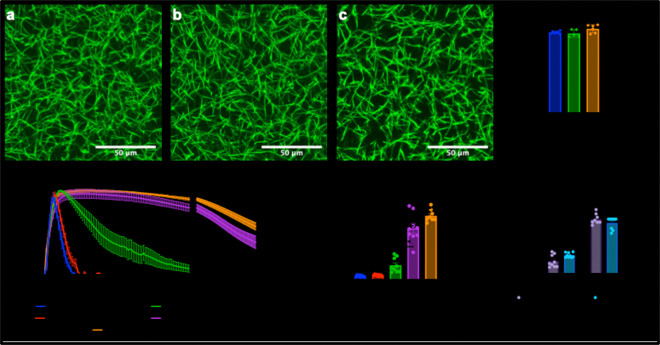
*PAI-1 does not affect structure but delays lysis. Confocal microscopy images of a) 0, b) 50, and c) 300 ng/mL. Scale bar is 50 microns. d) Percent area of fibers was measured from confocal images. e) Clot formation and fibrinolysis with increasing PAI-1 concentration.* f) Time to 50% lysis with increasing concentrations of PAI-1. g) Low concentrations of PAI-1 (i.e., 50 ng/mL) along with PC patients with low levels of PAI-1 were compared to high concentrations of PAI-1 (i.e., 300 ng/mL) along with PC patients with high levels of PAI-1 for time to 50% lysis. * p<0.06, ***** p<0.0001.

Since neither pore size, diameter, clotting factors, nor other fibrinolytic inhibitors correlated to hypo fibrinolysis of the subset of patients, we suggest that the main contribution to the resistance to lysis of PC patient fibrin clots *in vitro* was due to elevated levels of PAI-1 (Figure 3d, e). Our results showed that PAI-1 was the only protein or network feature that correlated to the delay in lysis (Figure 3, Supplemental Figure 4). Furthermore, samples with spiked PAI-1 emphasized the critical role the inhibitor plays in restricting lysis, especially at higher concentrations as comparable to seen with hypofibrinolytic PC patients, but not in clot formation and structure (Figure 4). PAI-1 binds to tPA to irreversibly inhibit tPA’s ability to bind to plasmin and ultimately cleave the fibrin fibers. It is known that diabetic patients have elevated PAI-1, which our study also found to be true [50] (Figure 3e). A recent study using PC cell lines and mouse models explored and found a direct relationship between PAI-1 levels and an increased risk of VTE [25]. They found that PAI-1 can be tumor-derived and restricts blood clot resolution. Despite this finding, very little research has further explored this relationship. In addition, it was shown that PAI-1 was a predictor of severity for COVID-19 [51] and even has a direct link to diabetes [52]. Increased levels of PAI-1 are not reduced by common prophylactic medications, such as anticoagulants, as they do not directly target PAI-1, rather they target enzymes contributing to clot formation. Thus, anticoagulants will not aid in the prevention of PAI-1-mediated hypo fibrinolysis. The same is true on the opposite end of the spectrum for patients who have a PAI-1 deficiency and are at a risk of bleeding. Nonetheless, PAI-1 is not a protein measured in routine blood tests.

Since all patients were already on heparin and some also on Eliquis (apixaban) or enoxaparin at the time of the blood draw, it was difficult to know the role anticoagulants play on resistance to fibrinolysis. Of note, despite all patients taking anticoagulants, the patients were still hypercoagulable with a shorter clotting lag time (Figure 1a, b). It has been shown that under most conditions, apixaban is able to aid in fibrinolysis as well as prevent the formation of a clot, suggesting that it plays a dual role in coagulation [38, 53]. It was found that heparin plays a similar role; in fact, it even can lead to adverse bleeding due to its initiation of the fibrinolytic system [54]. Anticoagulant treatment for PC patients is challenging due to the risk of bleeding, especially during surgery, but increased risk of clotting during chemotherapy treatments that lead to exaggerated hypercoagulable conditions. However, this was not the case in our PC patient population as the combination of prophylactic treatment did not correlate to hypercoagulability or hypo fibrinolysis. In other words, anticoagulant treatment did not affect fibrinolytic kinetics. Moreover, chemotherapy-driven hypercoagulability is driven by damage to endothelial cells and inflammation, increasing clotting, which may not be measurable in our *in* vitro setting [55]. This supports the idea that hypercoagulability of PC patients come from elevation of fibrinolytic inhibitors rather than clotting factors. Therefore, there is a need to develop an alternate treatment that targets the fibrinolytic pathway directly, such as PAI-1, rather than or in addition to prophylactic medications that aim to prevent clot formation.

Despite previous calls of action to develop treatments to target and inhibit PAI-1, little progress has been made [50, 56–58]. In the mid 2000s, tiplaxtinin was identified to be a selective and effective oral drug to inhibit PAI-1 and was thought to be ready to go to clinical trials [59, 60]. Unfortunately, it is still in the preclinical trials with little progress in two decades due to risk of bleeding [56]. TM5275 also showed some potential but has not advanced past the *in vitro* setting [61]. In addition to its role in regulating fibrinolysis, PAI-1 inhibitors were identified to reduce tumor growth [62]. An improved therapeutic with a similar mechanism to tiplaxtinin or TM5275 could play a dual role in preventing thrombotic complications as well as preventing cancer spreading or metastasis. Due to the high mortality rate and overall poor prognosis of PC, having this potential, personalized target is promising.

While we successfully characterized the clot structure profile and the mechanism of which patients had restricted fibrinolysis, there are limitations that provide new avenues for future work. First, our experiments were performed with platelet-poor plasma due to feasibility and reproducibility; however, future studies could use platelet-rich plasma or whole blood to incorporate the role of red blood cells and platelets. Since many relevant factors are released into or present in plasma, such as fibrinogen or PAI-1, our study gives a good representation of the clotting and fibrinolytic profiles, despite the lack of the presence of cells. Future work could explore potential inhibitors of PAI-1 to expand the research done on targeting it as a preventative action and treatment.

## CONCLUSION

Our results indicate that PAI-1 should be more routinely measured as a clinical marker as a risk of thrombosis and hypo fibrinolysis for PC patients. PC patients who also had diabetes or COVID-19 were more susceptible to higher levels of PAI-1, indicating a higher risk of VTE and a population that requires particular focus. There is a need to develop a PAI-1 inhibitor that can enhance fibrinolysis and reduce risk of VTE.

## Figures and Tables

**Table 1.** Patient demographics and co-morbidities, n (%).

	Total (n=17)	Survived (n=14)	Deceased (n=3)

**Mean age**	63.88	63.21	67

**Sex (male)**	11 (64.7)	9 (64.3)	2 (66.7)
**Comorbidities**	17 (100)	14 (100)	3 (100)
COVID-19	2 (11.8)	2 (14.3)	0 (0)
Diabetes	7 (41.2)	6 (42.8)	1 (33.3)
Hypertension	10 (58.8)	9 (64.3)	1 (33.3)
Coronary artery disease	3 (17.6)	3 (21.4)	0 (0)
COPD	3 (17.6)	3 (21.4)	0 (0)

**Chemotherapy**	5 (29.4)	3 (21.4)	2 (66.7)

**Anticoagulants**	17 (100)	14 (100)	3 (100)
Heparin	17 (100)	14 (100)	3 (100)
Enoxaparin	5 (29.4)	3 (21.4)	2 (66.7)
Eliquis	1 (5.88)	1 (7.14)	0 (0)
Combination	8 (47.0)	6 (42.8)	2 (66.7)

**Blood clotting events**			
Myocardial infarction	1 (5.88)	1 (7.14)	0 (0)
PVT	1 (5.88)	1 (7.14)	0 (0)
